# Exploring alarm fatigue among clinical nurses through a human factors engineering framework: a descriptive qualitative study

**DOI:** 10.3389/fpubh.2026.1896498

**Published:** 2026-08-19

**Authors:** Yuqi Liu, Jingxuan Zhang, Zimo Zhang, Li Tian, Panfeng Wang

**Affiliations:** 1 Liaoning University of Traditional Chinese Medicine, Shenyang, China; 2 Shaanxi University of Chinese Medicine, Xianyang, Shaanxi, China; 3 School of Nursing, Peking University, Haidian, Beijing, China; 4 Peking University Third Hospital, Department of Radiotherapy for Oncology, Haidian, Beijing, China

**Keywords:** alarm fatigue, alarm management, clinical alarm, human factors engineering, qualitative research

## Abstract

**Aim:**

To identify the factors affecting alarm fatigue among clinical nurses, using a human factors engineering model as the theoretical framework.

**Design:**

A qualitative descriptive design was employed.

**Methods:**

Purposive sampling was employed to recruit nurses and nurse managers from a large tertiary hospital in China. Semi-structured, in-depth interviews were conducted between August 2023 and February 2024. Using the human factors engineering model as the analytical framework, directed content analysis was conducted on the interview data.

**Results:**

In total 15 clinical nurses and 5 nurse managers were interviewed. Nurses reported factors associated with their personal factors, and their interactions with work environments and alarm devices. Dominant personal themes were linked to the ability of nurses to deal with alarms, awareness of alarms, responsibility, and emotional regulation. Environmental contributors comprised a supportive work atmosphere, open communication, noisy and crowded clinical wards, and patient conditions. Device-related issues such as inaccurate alarm levels, indistinguishable alarm sounds, and limited operability, also affected alarm fatigue.

**Conclusion:**

This qualitative study indicated that personal factors, work environments, and alarm device characteristics substantially affect alarm fatigue. These results suggest that alarm fatigue is modulated by several elements of the clinical system. A system-level approach to alarm management is crucial to reducing alarm fatigue, and should be prioritized in future efforts to improve clinical safety.

## 1 Introduction

Advances in medical technology have led to the widespread application of medical devices to monitor patients' conditions (1). Human factors engineering (HFE) is a systems-driven discipline that optimizes the fit among humans, work environment, and technology to improve safety, wellbeing, and performance (2). Clinical alarm systems require coordinated consideration of nurses, medical devices, and the clinical environment. As first-line direct healthcare providers, nurses constitute the primary recipients and responders to alarms from clinical medical devices, therefore, they are exposed to a considerable number of alarms produced by medical devices (3). Nurse managers are also important components of the alarm management environment, since they shape unit-level alarm culture, training, and alarm management practices (4).

In adult intensive care units, approximately 820 alarms are generated by devices per patient each day, and this number can increase up to 1,503 alarms (5). Previous studies have reported that over 80% of alarms are non-actionable, meaning they do not require clinical intervention (6, 7). Frequent false alarms have been reported as a significant barrier to effective alarm management (8). Repeated exposure to excessive or non-actionable alarms can lead to alarm fatigue (AF), a phenomenon in which healthcare staff become desensitized to alarm signals and may delay or fail to respond appropriately (9). Several studies have shown that AF is relatively prevalent among nurses (10, 11). In China, studies among ICU nurses have similarly reported moderate levels of alarm fatigue. Wang et al. reported a mean alarm fatigue score of 18.76 ± 6.85 on a 35-point scale (12), while Liu et al. reported a mean score of 26.38 ± 5.32 among rotating night-shift ICU nurses on a 44-point scale (13).

Alarm fatigue may compromise patient safety (9). Moreover, nurses, as the staff directly caring for patients and reacting to alarms, experience an increased burden of alarm fatigue, leading to errors, burnout, and poor sleep quality (14–16). In its 2025 National Patient Safety Goals, the Joint Commission has emphasized the need for “reducing patient harm associated with clinical alarm systems” (17). Despite these concerns, the number of clinical alarms has continued to increase. Therefore, alarm fatigue is a safety and quality problem, and addressing this challenge is vital for ensuring patient safety and enhancing the wellbeing of nurses.

Understanding the factors contributing to alarm fatigue is essential for effective intervention. Although many quantitative studies have explored the factors affecting alarm fatigue and alarm management, such as workload, work experience, alarm knowledge, and training, the results remain inconsistent (11, 15, 18). Meanwhile, existing studies have limited ability to identify the mechanisms that underlie these phenomena, including environmental factors and the design of medical devices related to alarm fatigue. A deeper insight into these mechanisms is essential for developing system-level interventions to improve alarm management and the work environments of nurses. Furthermore, a few studies have explored the contributors to alarm fatigue from the perspective of nurses, especially among nurses working in diverse units rather than those solely working in intensive care units.

HFE is an approach that starts from the psychological and physiological characteristics of humans and aim to explore and optimize human-related interactive systems (19). Therefore, based on HFE theory, this study adopted a qualitative research design to explore the factors affecting alarm fatigue among clinical nurses. The study aimed to reduce alarm fatigue, improve patient safety and work experience of nurses, and provide a theoretical foundation for interventions, thereby improving alarm fatigue management in the clinical settings.

## 2 Methods

### 2.1 Study design

This study employed a qualitative descriptive design to explore the factors affecting alarm fatigue among clinical nurses, using semi-structured one-on-one interviews.

### 2.2 Theoretical framework

HFE is the study of interactions among humans, machines, and their working environments. It aims to so design systems that accommodate human physiological and psychological characteristics while addressing safety and efficiency goals (19).

Currently, in the healthcare sector, HFE is primarily applied in three areas: the development and improvement of medical devices and environments, the implementation of information technology in the healthcare system, and the enhancement of patient safety while decreasing the workload of healthcare personnel (20).

**Figure 1:**
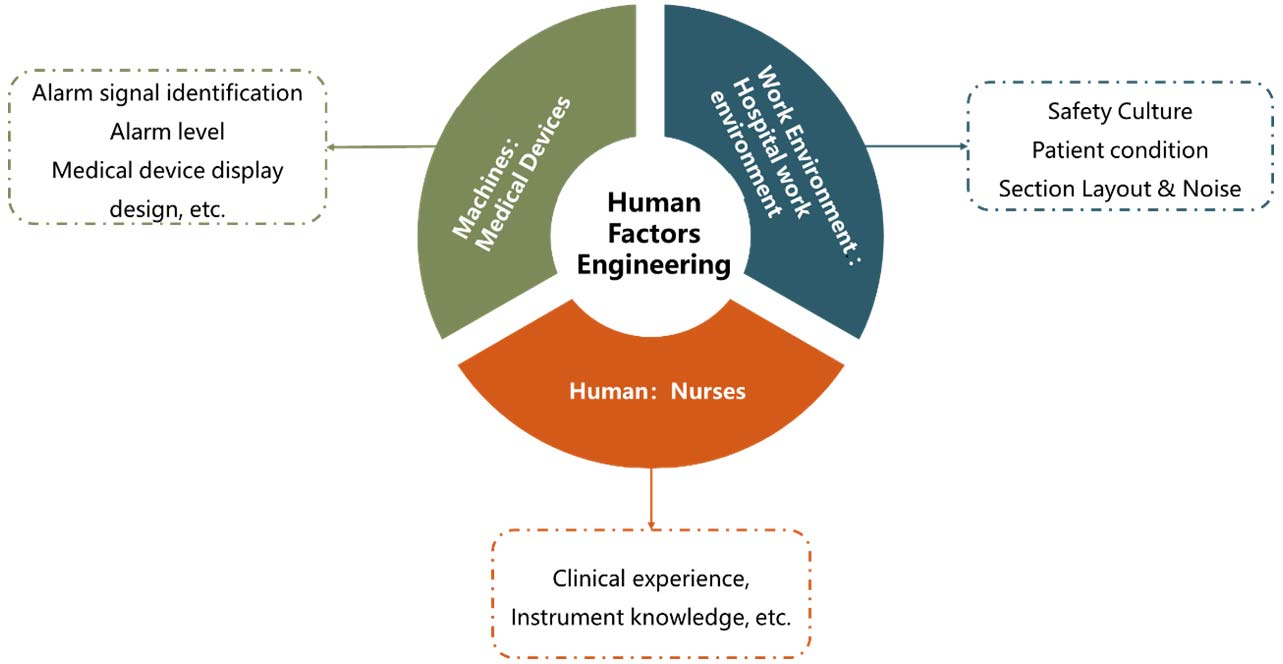
Human Factors Engineering framework for exploring factors affecting alarm fatigue among clinical nurses

HFE emphasizes that humans are the starting point of design and primarily studies the correlations between humans and other elements. In alignment with this perspective, the study starts from the nurses themselves and employs semi-structured interviews to deeply explore the factors affecting alarm fatigue of nurses across the three domains of themselves, machines, and their work environment (Figure 1).

### 2.3 Study sample and participants

A purposive sampling method was utilized to recruit nurses and nursing managers whose work involved clinical alarms, because they could provide information directly linked to the aims of this study. Based on the clinical experience of the research team and team discussion, clinical units in which alarm-related work was routine were first identified. Participants were recruited from a large tertiary hospital with more than 1,800 beds in Beijing, the capital city of China. The alarm-related clinical units included inpatient units, intensive care units, emergency units, anesthetic units, and the hemodialysis center.

The first author contacted the nursing manager of each unit, explained the purpose and procedures of the study, and asked the nursing manager to help distribute study information to potentially eligible nurses. Nurses who were interested in participating voluntarily and met the inclusion criteria were then contacted by the first author for further explanation and interview arrangement.

To capture diverse alarm-related experiences, recruitment aimed to include participants with variation in clinical unit, professional role, professional title, educational level, and length of clinical experience. The following inclusion criteria were applied to recruit nurses: (i) being a registered nurse; (ii) working in the hospital for at least the last 6 months; and (iii) clinical work involved alarms. The 6-month working experience was to ensure that the participating nurses had sufficient clinical exposure and familiarity with routine clinical alarm work. The following inclusion criteria were applied to recruit nursing managers: (i) working in clinical departments; (ii) have management experience for at least 1 year; and (iii) management work involved alarm management. The exclusion criteria were as follows: (i) visiting or temporary training nurses; (ii) nurses absent from the department during the study period because of leave, external training, or other reasons; and (iii) administrative nursing managers who did not work in clinical departments. Participant recruitment continued until data saturation was achieved, signifying no emergence of new information. Data saturation was confirmed after the 16th interview, when no substantially new codes or themes emerged. Four additional interviews were conducted to confirm data saturation. The final sample included clinical nurses, teaching nurses, and nurse managers from different alarm-related units, as shown in Table 1.

**Table 1:** Descriptive statistics of participant characteristics (*N* = 20).

Participant ID	Age	Gender	Education	Position	Length of working experience	Job duty	Work departments
N1	42	Female	College education	Primary (nurse practitioner)	22	Clinical nurse	Inpatient unit
N2	43	Female	Undergraduate education	Moderate	22	Teaching nurse	Inpatient unit
N3	27	Female	Undergraduate education	Primary (nurse practitioner)	2	Clinical nurse	Emergency
N4	28	Female	Postgraduate education	Primary (nurse practitioner)	4	Clinical nurse	ICU
N5	24	Female	Undergraduate education	Primary (nurse)	2	Clinical nurse	Inpatient unit
N6	42	Female	Undergraduate education	Moderate	19	Teaching nurse	Inpatient unit
N7	23	Female	Undergraduate education	Primary (nurse)	1	Clinical nurse	ICU
N8	24	Female	College education	Primary (nurse practitioner)	3	Clinical nurse	ICU
N9	27	Male	Undergraduate education	Primary (nurse practitioner)	6	Teaching nurse	ICU
N10	37	Male	Undergraduate education	Moderate	15	Teaching nurse manager	ICU
N11	31	Female	Undergraduate education	Primary (nurse practitioner)	10	Teaching nurse	ICU
N12	42	Female	Undergraduate education	Moderate	21	Nurse manager	ICU
N13	27	Female	Postgraduate education	Primary (nurse practitioner)	2	Clinical nurse	Emergency
N14	45	Female	Undergraduate education	Moderate	25	Nurse manager	Anesthetic unit
N15	33	Female	Postgraduate education	Moderate	10	Teaching nurse	Anesthetic unit
N16	40	Female	Undergraduate education	Moderate	18	Teaching nurse	Anesthetic unit
N17	43	Female	Undergraduate education	Moderate	25	Teaching nurse	Hemodialysis center
N18	54	Female	Undergraduate education	Moderate	36	Nurse manager	Hemodialysis center
N19	26	Female	Undergraduate education	Primary (nurse practitioner)	6	Clinical nurse	Hemodialysis center
N20	39	Female	Postgraduate education	Vice senior	15	Nurse manager	Emergency

### 2.4 Interview guide

With reference to previous domestic and international studies on alarm fatigue, the semi-structured interview guide was developed based on the aims of this study and the theoretical framework. The draft guide was reviewed by qualitative research experts and discussed within the research team. Subsequently, it was revised after pilot interviews and finalized for use in formal interviews. The guide included the core questions listed in Table 2. The interview questions were not limited to what is presented in Table 2 and were based on the thoughts of the interviewees, enabling them to fully express their perspectives.

**Table 2:** Semi-structured interviews include the questions.

Nurses	Nursing managers
1. Have you experienced alarm fatigue in your clinical work? Could you describe that experience?	1. Have you observed alarm fatigue among nurses in your department? Could you describe what you have noticed?
2. What do you think leads to your feelings of alarm fatigue during your work? (Environmental, Personal, medical device)	2. What do you think causes nurses' alarm fatigue during their work? (Environmental, Personal, medical device)

### 2.5 Ethical considerations

This study was conducted following the Declaration of Helsinki and was approved by the Ethics Committee of the ‘Peking University Third Hospital'. The work team ensured adherence to the ethical principles. All participants signed informed consent forms before participation. To protect confidentiality, in reporting the results, each participant was given a pseudonym and edited details about their department.

### 2.6 Data collection

Between August 2023 and February 2024, semi-structured interviews were conducted with the participants.

At the beginning of each interview, the purposes and the contents of the survey were explained, and the recording began after obtaining informed consent. The interviews were organized around questions (Table 2) and conducted in the office of each department. The interviews were held by a single interviewer and lasted from 30 to 58 min (*M* = 39.25, SD = 8.67).

During interviews, probing questions were applied to explore the connotations and influencing factors of alarm fatigue, such as “Could you explain more about that?” and “What do you mean?” The interviews were conducted by the first author, who had a nursing background and was trained in qualitative research methods and semi-structured interviewing before data collection. No prior interaction existed between the interviewer who conducted the interview and the participants. This minimized the effect of the interviewer during the interviews. The interviews were conducted face-to-face in a private office in each department. The Tencent meeting was used only as an audio-recording and transcription-support tool after obtaining the consent of the participants. The recordings were transcribed verbatim by the first author and a research assistant for further analysis. Data collection and data analysis were conducted simultaneously.

### 2.7 Rigor

Guidelines for standards for reporting qualitative research (SRQR) were followed in this study (**Appendix S1**: A corresponding table between checklist items and manuscript content is included in **Appendix S1**).

The criteria of credibility, dependability and confirmability were employed to establish study trustworthiness (21). Credibility was strengthened by obtaining data on nurses with different characteristics. Before data collection, the interviewer received training in qualitative research methods and reviewed relevant qualitative research literature (22, 23). During the interviews, the interviewer avoided leading questions, listened carefully, gave participants sufficient time to reflect, and adopted clarification and probing questions to confirm the meanings of the participants. Although formal member checking was not conducted, these procedures ensured that the participants' views were accurately understood during data collection. Dependability was enhanced through independent coding by two researchers and repeated discussions of codes and themes within the research team until reaching a consensus. A de-identified coding record was retained as an audit trail to document the development from initial codes to final themes. Confirmability was promoted through reflexive diary writing and research team discussions to minimize the effects of individual assumptions on data interpretation. The first author's clinical experience in responding to alarms and the use of the HFE framework were recognized as potential influences on interpretation, because familiar alarm-related experiences could be taken for granted and the predefined framework could orient analysis toward the three HFE domains. These reflections were recorded in the reflexive diary and revisited during team discussions on coding and theme development.

### 2.8 Data analysis

A directed content analysis was conducted to deductively analyze qualitative data, using HFE as the theoretical framework. First, transcripts were deconstructed into meaningful units and codes. Second, the researcher was fully immersed in the material and reads it carefully and repeatedly. The HFE framework was used as the predefined coding framework to categorize the units. During coding, the researcher remained open to data possibly not fitting the predefined domains. Codes that were unclear or did not immediately fit the matrix were reviewed against the original interview data and discussed within the research team. Finally, similar sentences and paragraphs were conceptualized and coded. Thereafter, similar codes were clustered into appropriate categories to form themes and subthemes. Throughout the study process, the researcher recorded her reflections by writing memos. These memos also served as a constant reflection on the entire study, helping to understand coding, perspectives, and ideas for the next steps of this study.

## 3 Results

### 3.1 Demographic characteristics of participants

In total, 15 clinical nurses and five nurse managers participated in the interviews. The participants were from various clinical units, including the inpatient unit, ICU, emergency, anesthetic unit, and hemodialysis center. The characteristics of participants are summarized in Table 1.

### 3.2 Thematic analysis findings

**Figure 2:**
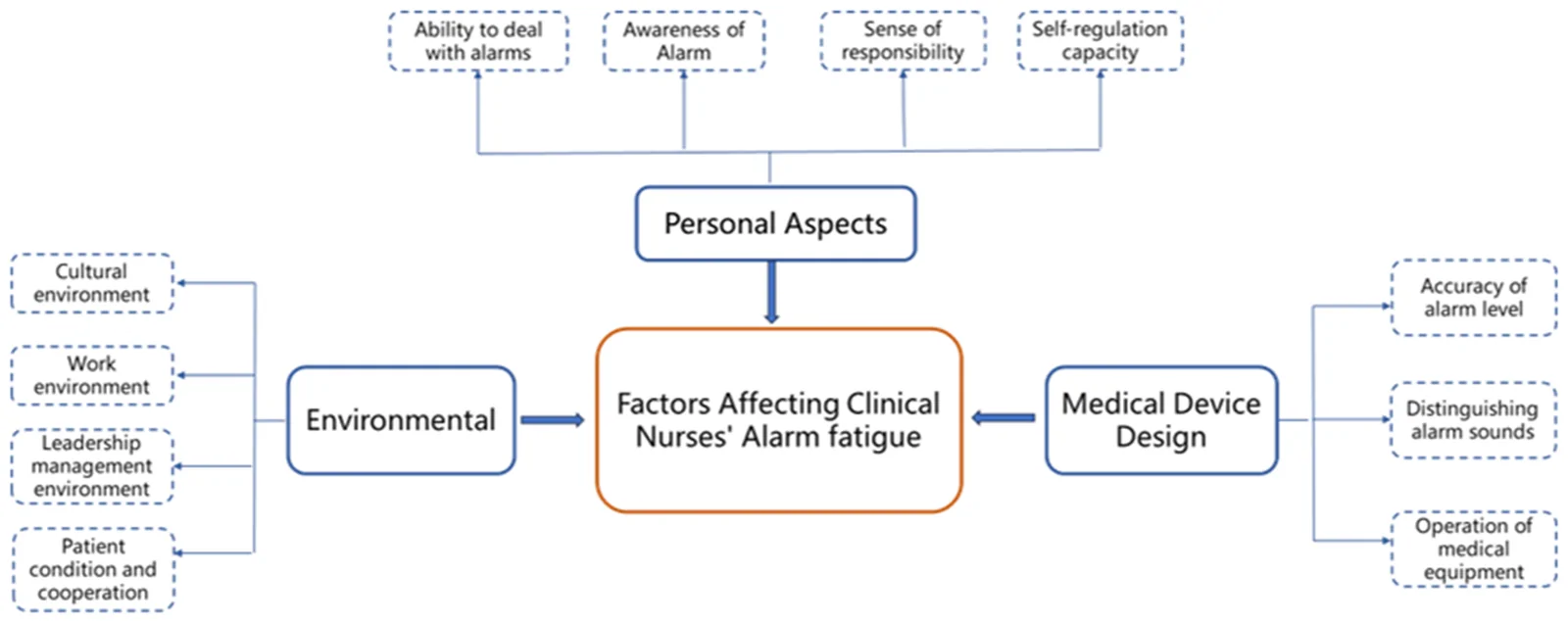
Themes and subthemes of factors affecting alarm fatigue among clinical nurses.

Thematic analysis identified three major themes and eleven 11 subthemes that reflect the factors affecting the alarm fatigue of nurses within personal, environmental, and medical device design domains. Figure 2 presents the themes and subthemes.

#### 3.2.1 Theme 1: personal aspects

Participants described personal factors affecting their experience of alarm fatigue. They mentioned that individual knowledge and clinical experience affected their perception of alarms. Some nurses reported that frequent exposure to alarms led to mental overload and anxiety, which subsequently reduced their responsiveness. Differences in responsibility and emotional regulation also affected the perception of alarm fatigue among nurses.

Five subthemes were included in personal aspects (Table 3).

**Table 3:** Subthemes, codes and verbatims of theme 1: personal aspects.

Subthemes	Codes	Verbatims
Ability to deal with alarms	Theoretical knowledge about alarms	N6: Then, like some of the notes we take with (medical equipment), ……, it's not systematic training, it's something that happens and we find out that it has an impact……. N18: Sometimes I really don't understand what some of the uncommon (alarms) mean. ……, don't know where to cancel the alarms.
	Clinical experience	N14: “I think the speed of response is based on your clinical experience, you know what's important,…….” N9: “Lower seniority nurses may have encountered fewer (alarms) situations, not as experienced, and a lot of situations that they don't know how to respond to and won't be responded to in a timely manner.”
	Confidence in responding to alarms	N5: “Maybe it involves all that kind of electronics, I don't know a lot, sometimes I don't dare to deal with it, …….”
Awareness of Alarm	Recognizing the dangers of alarms	N12: “Some of young nurses ……, and in her perception it might be just the loud alarms that might need to be respond to.” N20: “Adverse events of alarms seem to be rare and sometimes I don't pay that much attention to alarms”
	Perception of alarms importance levels	N15: “It's also not a particularly urgent life-threatening alarm that I wouldn't fail to deal with for a while” N17: “It is rare to have a delayed response for not dealing with alarms that cause serious consequences.”
Sense of responsibility	Proactive in responding to alarms	N2: “Responding to alarms has something to do with responsibility……. Not paying attention to you as a job, she might not pay attention to these (alarm) issues.” N9: “Some people will turn it (alarms) off, just this any kind of people.” N20: “Transient changes can clear automatically, and some people just don't deal with them. But when I hear that sound, sitting here, I still feel uneasy, so once that sound appears, my first reaction is to clear the alarm.”
Self-regulation capacity	Reducing stress and anxiety of clinical alarms	N11 and N19: “Sometimes with 2–3 days off, ……, so that go back to the section in good shape (to respond to alarms)” N11: “I feel that talking with good colleagues and good friends helps me relax and relieve stress, so that I feel less anxious and the alarm sounds no longer bother me so much.”

##### 3.2.1.1 Ability to deal with alarms

The ability to deal with alarms suggests adequate alarm knowledge and confidence to enable clinical nurses to perceive the level of importance of alarms, which enables them to respond to alarms faster and more safely.

Nurses considered that they lacked the knowledge to respond to alarms and had no training in alarms. They also highlighted clinical experience would help them to deal with alarms and recognize alarm levels of importance.

##### 3.2.1.2 Awareness of alarm

Nurses' awareness of the information conveyed by clinical alarms, specifically the importance, urgency, and potential risk represented by the alarms, can affect their response speed and handling methods. Most nurses stated that the speed of response depends on the possible consequences of the alarm.

##### 3.2.1.3 Sense of responsibility

A sense of personal responsibility reflects concern for patient safety and a conscientious approach to work, leading to more proactive action. Nurses with a high level of responsibility will respond to various clinical alarms timely, to check for patient deterioration.

##### 3.2.1.4 Self-regulation capacity

Self-regulation is the ability to manage one's time, energy, and focus while reducing psychological stress and anxiety. The participants perceived that adequate rest and talking to colleagues and friends can help them adjust themselves to a better psychophysiological state, thereby preventing fatigue.

#### 3.2.2 Theme 2: environmental

Our findings showed that the work environment plays a significant role in the development of alarm fatigue. A supportive work atmosphere within the department, especially an environment encouraging open communication and a positive attitude toward mistakes, was found to modulate their perception of alarm fatigue. Besides, noisy and crowded clinical wards, heavy workloads, and patients' conditions were reported to exacerbate alarm fatigue.

Four subthemes were included in the environmental theme (Table 4).

**Table 4:** Subthemes, codes and verbatims of theme 2: environmental.

Subthemes	Codes	Verbatims
Cultural environment	Friendly work environment	N11: “(Leaders)……, you live in a very loving environment, you're going to a state of being better in every way(including responding to alarms).”
		Open communication	N8: “If we have clinical alarm problems to feed back to him [the leader], he can give us positive feedback.”
	Positive attitude toward clinical errors and risks	N11: “……I have a problem at work, maybe about clinical alarms, ……. When I talk about it, I want my leaders to give me encouragement and improvement, not that they keep criticizing…….”
	Teamwork	N19: “Sometimes I don't notice an alarm, but other teachers will definitely notice it and respond in time, it's true!” N6: “I might have a communication powerlessness and between other teams. For example, if the value is a little bit higher, ……by notifying them(doctors), you know that the answer you get not change, ……alarms all the time, and we might turn it off or not be responding to it.”
Workspace dynamics	Departments layout structure	N3: “There's also the issue of the distance of the wards, the alarm on your side ……might not be heard in this case.” N13: “There are times when ……you patient is pushed into that place (corner) where you can't see it, then ……that corner is ringing, but I don't know specifically if it's this bed or that bed.”
	Noise pollution in the departments	N1: “Particularly noisy states are definitely affected and will probably go unheard or less audible (alarms).”
Leadership management environment	Nurse/patient ratio	N1: “I'm sure I'll manage it when I'm not busy yah, I'm definitely quicker……. But I manage several patients, several alarms at the same time, ……prioritize alarms related to emergency. I can't split up.”
	Alarm management program	N18: “Follow the operating procedures, step by step.……. It will greatly avoid some of the problems……, because there are some problems that it alarms.”
	Division of responsibility for responding to alarms	N7: “When it comes to alarm, you really can't say who's to blame if something goes wrong.”
	Alarm training	N1: “My English might not be that great, ……I'll need some training or instruction beforehand!”
	Leadership oversight	N5: “Our manager mentioned that if he emphasizes alarms during the morning shift, everyone might start responding to them more promptly.”
	Development of technology	N16: “Our record sheets are fully integrated, and alarm records are imported straight from the system, which really saves us a lot of time.”
Patient condition	Information and signs of the patient	N10: “I found that this patient set off an alarm, but …… nothing out of the ordinary occurred—they remained stable. So I was able to delay responding to it. For instance, if I have other patients who need immediate care, I might go attend to them first.” N9: “You have to understand this patient's condition before you can make appropriate settings for their monitoring.”

##### 3.2.2.1 Cultural environment

The philosophy, rules, and regulations within an organization determine the way the organization works and shape its atmosphere and style.

The nurses stated that the concern shown by department managers and the creation of a friendly work environment positively affected their alarm fatigue. The nurses also indicated that positively confronting clinical errors and risks increases their motivation to respond to alarms.

Most nurses stated that collaboration among colleagues helped expedite the resolution of alarm-related issues. In rush hours, mutual assistance can prevent alarm fatigue. Efficient communication between departments is also crucial for promptly addressing technical issues related to alarms, which in turn affects alarm response.

##### 3.2.2.2 Workspace dynamics

The dynamics of the workspace in the unit significantly affect alarm fatigue. Most nurses felt that the layout of the unit, excessive distances, and obstructed equipment do not allow the prompt identification and localization of alarms, thereby affecting the efficiency of alarm response.

The noise levels can affect alarm recognition by nurses. Some nurses have reported that excessive noise in the unit, including conversations and alarms, may interfere with alarm detection and delay their response.

##### 3.2.2.3 Leadership management environment

The nurse-patient ratio and the division of labor significantly affect the speed of alarm response. The participants indicated that in case of a high workload and an insufficient number of staff, they will prioritize alarms related to emergency patients. Establishing alarm management programs and standardizing equipment operation processes can minimize the number of alarms, especially false and non-actionable alarms.

Alarm-related issues involve multiple departments. Nurses hoped that responsibility for alarm handling could be implemented so that each member of the organization would recognize their roles and responsibilities, which can improve the alarm response rate.

##### 3.2.2.4 Patient condition

Most nurses emphasized that the severity of a patient's condition affects the speed of alarm response. They paid more attention to alarms for patients with serious and emergency conditions. Furthermore, understanding changes in a patient's condition and assessing the association of alarms with immediate potential harm are key factors determining how quickly nurses should respond to alarms.

#### 3.2.3 Theme 3: medical device design

Participants described several device-related issues that affected their experience of alarm fatigue. These issues included inaccuracies in alarm levels, difficulty in distinguishing alarm sounds, and limited operability of alarm settings.

Three subthemes were included in medical device design (Table 5).

**Table 5:** Subthemes, codes and verbatims of theme 3: medical device design.

Subthemes	Codes	Verbatims
Accuracy of alarm level	Repeating the same alarms	N9: “The alarms go off way too often, ……The first few times might be false alarms or……, but eventually, there could be a real emergency that leads to bad outcomes.”
	Too much nonactionable alarms	N10: “……, with the ventilator, a patient might cough or choke, which triggers an alarm.……. But by the time we get there, the alarm might have stopped, so gradually I might stop paying attention to it.”
Distinguishing alarm sounds	Confusing the alarms sounds of different devices	N11: “The new monitors have alarm sounds exactly like our infusion pumps. We just can't tell the alarm sounds apart.”
	Alarm sounds indicate alarm levels of severity	N4: “It have three levels of alarms. Usually, the high-level alarms keep going, the sound is really loud and very sharp. ……you have to get there quickly.”
Operation of medical equipment	User-friendly design	N17: “……ventilators aren't just about what patients need—they're also designed to be user-friendly for the operators.” N12: “For example, a patient died over the weekend and their heart rate became zero, so the nurse might turn off the alarm. Then a new patient is admitted, and when the machine is turned on it stays in the previous state—the alarm is off—but they don't know it. That's very dangerous.”
–	More modification powers	N17: “……in actual clinical settings, the machine's functions don't really match up because there's a lot of individual differences, and we can't adjust the threshold settings ourselves.”

##### 3.2.3.1 Accuracy of alarm level

The accuracy of alarm levels refers to the medical equipment's ability to correctly represent immediate potential harm. Inaccurate alarm levels can decrease nurses' trust in alarms, contributing to alarm fatigue.

Nurses highlighted that medical equipment often issues the same repeated alarms, and sometimes these alarms do not reflect any change in the patient's condition.

The slightest change in value may activate the instrumental alarm, and currently available alarms are too sensitive, leaving nurses with an overload of daily alarms. Many of these alarms reflect transient changes that do not represent actual emergencies.

##### 3.2.3.2 Distinguishing alarm sounds

When different instruments produce the same sound, a delayed response to alarms or an incorrect response can be expected. Nurses stated that not being able to distinguish between alarm sounds can result in their confusion.

##### 3.2.3.3 Operation of medical equipment

Nurses described that the current medical equipment page design is too complex and that alarm settings are not automatically restored when a new patient is connected. In addition, nurses are provided with very few adjustable parts, making it difficult to set up alarms for patients with specific conditions and leading to a large number of non-actionable alarms.

## 4 Discussion

In this qualitative study, clinical nurses detailed several factors affecting alarm fatigue. The dominant factors included personal factors, environmental factors, and medical device factors. Alarm fatigue was found to originate from a persistent mismatch between the personal circumstances of nurses, work environments, and the technologies of devices.

A lack of knowledge about managing and responding to alarms can lead to inappropriate alarm settings or delayed responses, which can increase the likelihood of alarm fatigue. This is consistent with previous reports showing that enhanced alarm knowledge can reduce alarm fatigue (4). This finding is also supported by recent quantitative evidence indicating that prior alarm training is a significant independent predictor of better alarm management practices, whereas insufficient training may contribute to alarm desensitization and delayed responses (8, 18). Sufficient alarm knowledge and experience allow nurses to quickly and accurately understand the meaning of alarms and adopt appropriate methods to resolve them (24). The present study indicated that nurses with greater clinical experience are more successful in alarm management, suggesting that experience protects against alarm fatigue (3). A higher level of attention and awareness to clinical alarms, and a greater emphasis on assessing the severity of alarms, were found to improve response speed. These findings are consistent with those of a prior qualitative study (25). Alarm related knowledge and clinical experience should not be regarded only as individual attributes. They are also shaped by the organizational environment for training, peer learning, and feedback. Therefore, supportive learning environments should strengthen alarm management practices among nurses and prevent alarm fatigue.

Consistent with our findings, Movahedi et al. reported that personal responsibility and self-regulation ability also affect alarm fatigue (26). Nurses with a strong sense of responsibility are more respectful of life and perform their duties conscientiously. Besides, adequate rest helps restore physical energy and mental state, enabling individuals to maintain sufficient concentration when facing emergencies and reducing psychological stress and anxiety. Environmental conditions may affect the psychological state and self-regulation of nurses, thereby controlling their alarm fatigue. Severe psychosocial burdens and environmental stressors may impair the self-regulation and cognitive capacity of nurses while processing clinical alarms. The emphasizes that organizational interventions are need to address the underlying psychological distress (27, 28). A positive organizational culture improves attitudes, practices, and patient safety (29). A friendly work environment, open communication, effective teamwork, and supportive leadership may help reduce alarm fatigue by providing psychological support, facilitating timely actions, and enabling nurses to manage clinical alarms (30). This study indicated that a supportive management environment (e.g., improved nurse-to-patient ratio) enables clinical nurses to manage alarms more effectively. Similar findings were reported by Newton et al. (31). An insufficient number of staff forces nurses to prioritize critically ill patients, potentially delaying responses to other alarms and increasing the risk of alarm fatigue. Excessive noise and physical layout further undermined the ability of nurses to detect and localize alarms. Similarly, Bruder et al. (32) reported that, noise diverts attention required for completing critical tasks. The layout and structure of the department may make it more difficult to localized and respond to alarms, which is consistent with previous findings (3, 33). Patients' conditions determine the speed of alarm handling, as nurses offer patient-centered care. Similarly, another study (25) reported that, patients' conditions affect the degree of attention paid by nurses to patient alarms, thereby affecting the speed of their response. Furthermore, the individual behaviors of some patients may also affect alarm fatigue. For example, older adult patients, children, or those with a history of mental illness may face difficulties in cooperating with nursing procedures, which can result in errors and nonactionable alarms. These findings suggest that leaders should develop personalized and system alarm management strategies to minimize non-actionable alarms, thereby alleviating alarm fatigue.

The design of medical devices also affects alarm judgment and management behaviors. Ambiguous alarm information, inaccurate alarm levels, difficult-to-distinguish alarm sounds, and non-intuitive interfaces may limit the ability of nurses to adjudicate alarm priority and manage alarms effectively, thereby contributing to alarm fatigue. Alarm levels that accurately reflect patients' conditions can enable nurses to prioritize important tasks, allocate their time efficiently, and reduce the burden of alarm fatigue. Fang et al. (34)demonstrated similar improvements in efficiency and workload through a big-data–based optimization of nighttime ICU alarms. Improving the design of alarm sounds can enhance the response speed (32). Effective differentiation of alarm sounds enables responders to quickly recognize the issues they are currently facing and avoid confusion, thereby preventing alarm fatigue. An intuitive and user-friendly interface allows nurses to understand alarm information and device status more rapidly, contributing to faster and safer responses (35, 36). In addition, the manipulation of medical devices by nurses represents an influencing factor (37). Given the complexity and individual variability in clinical conditions, nurses require sufficient authority to adjust alarm thresholds. When necessary, nurses may temporarily silence non-critical alarms to avoid unnecessary interruptions.

Participants' accounts also suggested that alarm-related experiences varied across clinical unit contexts. In intensive care units, where multiple devices are used simultaneously, participants highlighted frequent non-actionable alarms and difficulty distinguishing alarm sounds. In emergency units, crowded spaces and patients' movement between locations appeared to make alarm detection and localization more difficult. In anesthetic units, clinical experience and comparatively integrated information systems appeared to reduce some of the workload associated with alarm management. Similar context-specific patterns have been reported in a multicenter study of monitor alarms in emergency departments (33) and in intensive care units, where alarm optimization has often been approached through device and data-driven strategies (34). Given the small number of participants in each unit, these observations should be interpreted as contextual rather than comparative. Nevertheless, they suggest that priority targets for alarm management may differ by setting.

Therefore, interventions to reduce alarm fatigue should not focus on a single factor alone, but should combine alarm-related training, supportive work environment strategies, and improvements in medical device design and application.

### 4.1 Limitations and future research directions

This study faced several limitations. First, the findings were derived from a qualitative sample from one large tertiary hospital, therefore, they may not be generalizable to other settings. Although participants were included from multiple clinical units, including inpatient, ICU, emergency, anesthetic, and hemodialysis departments, future studies should include nurses from different regions, hospital levels, or specialties and examine unit-specific differences to further enrich the themes and strengthen the explanatory framework. Second, the study focused solely on the perspectives of nurses and did not incorporate the viewpoints of other stakeholders, such as physicians, biomedical engineers, or administrators. This may limit the comprehensiveness of the findings. Third, nurses experiencing more pronounced alarm fatigue might have more incentives to participate, introducing selection bias. Finally, this study used directed content analysis and an interview guide based on the HFE framework, which may have shifted the results toward predefined theoretical categories. The researchers remained open to data that did not fit the initial coding framework. Future studies should combine framework-guided and more inductive approaches to further explore alarm fatigue experiences. They should also integrate multiple perspectives, combine quantitative research, and conduct mixed-methods studies to validate and extend these findings.

## 5 Conclusions

Alarm fatigue among clinical nurses was affected by the interaction between nurses' personal factors, work environment, and medical device design. Healthcare managers should develop system-level alarm management interventions that integrate psychological support, organizational improvements, and device-design optimization. Establishing work environments that support effective alarm management is essential for reducing alarm fatigue, protecting the wellbeing of nurses, and sustaining high-quality clinical care and patient safety.

## Data Availability

The datasets presented in this article are not readily available because the original recordings were destroyed after the data analysis was completed. Requests to access the datasets should be directed to yuqi liu lyqhl98@163.com
