# Supplementary material for: Exploring alarm fatigue among clinical nurses through a human factors engineering framework: a descriptive qualitative study
Source: Front Public Health. 2026 Aug 19;14:1896498. doi: 10.3389/fpubh.2026.1896498 (PMC13534094; doi:10.3389/fpubh.2026.1896498)
Supplement: Supplementary file 1 [file Table_1.pdf]

Table SRQR

Topic	NO.	Item	Reported on / Location in manuscript
**Title and abstract**			
Title	1	Concise description of the nature and topic of the study identifying the study as qualitative or indicating the approach (e.g., ethnography, grounded theory) or data collection methods (e.g., interview, focus group) is recommended	Title page
Abstract	2	Summary of key elements of the study using the abstract format of the intended publication; typically includes objective, methods, results, and conclusions	Abstract
**Introduction**			
Problem formulation	3	Description and significance of the problem/phenomenon studied; review of relevant theory and empirical work; problem statement	Introduction
Purpose or research question	4	Purpose of the study and specific objectives or questions	Introduction
**Methods**			
Qualitative approach and research paradigm	5	Qualitative approach (e.g., ethnography, grounded theory, case study, phenomenology, narrative research) and guiding theory if appropriate; identifying the research paradigm (e.g., positivist, constructivist/interpretivist) is also recommended	2.1.1, Theoretical Framework
Researcher characteristics and reflexivity	6	Researchers' characteristics that may influence the research, including personal attributes, qualifications/experience, relationship with participants, assumptions, or presuppositions; potential or actual interaction between researchers' characteristics and the research questions, approach, methods, results, or transferability	2.5,Data Collection; Section;<br>2.6, Rigor
Context	7	Setting/site and salient contextual factors; rationale†	2.2,Study sample and participants
Sampling strategy	8	How and why research participants, documents, or events were selected; criteria for deciding when no further sampling was necessary (e.g., sampling saturation); rationalea	2.2,Study sample and participants
Ethical issues pertaining to	9	Documentation of approval by an appropriate ethics review board and participant consent, or explanation for lack thereof; other confidentiality and data security issues	2.4,Ethical Considerations
Data	10	Types of data collected; details of data collection	2.5,Data

collection methods		procedures including (as appropriate) start and stop dates of data collection and analysis, iterative process, triangulation of sources/methods, and modification of procedures in response to evolving study findings; rationalea	Collection
Data collection instruments and technologies	11	Description of instruments (e.g., interview guides, questionnaires) and devices (e.g., audio recorders) used for data collection; if/how the instrument(s) changed over the course of the study	2.3, Interview Guide;<br>2.5,Data Collection
Units of study	12	Number and relevant characteristics of participants, documents, or events included in the study; level of participation (could be reported in results)	3.1, Demographic Characteristics of Participants; Table 2
Data processing	13	Methods for processing data prior to and during analysis, including transcription, data entry, data management and security, verification of data integrity, data coding, and anonymization/ de-identification of excerpts	2.5,Data Collection; 2.7,Data Analysis
Data analysis	14	Process by which inferences, themes, etc., were identified and developed, including researchers involved in data analysis; usually references a specific paradigm or approach; rationale†	2.7,Data Analysis
Techniques to enhance trustworthines	15	Techniques to enhance trustworthiness and credibility of data analysis (e.g., member checking, audit trail, triangulation); rationale†	2.6, Rigor
**Results/Findings**			
Synthesis and interpretation	16	Main findings (e.g., interpretations, inferences, and themes); might include development of a theory or model, or integration with prior research or theory	3.2, Thematic analysis findings; Figure 2
Links to empirical data	17	Evidence (e.g., quotes, field notes, text excerpts, photographs) to substantiate analytic findings	3.2, Thematic analysis findings;<br>Table 3-5
**Discussion**			
Integration with prior work, implications, transferability, and contribution(s) to the field	18	Short summary of main findings; explanation of how findings and conclusions connect to, support, elaborate on, or challenge conclusions of earlier scholarship; discussion of scope of application/generalizability; identification of unique contribution(s) to scholarship in a discipline or field	4, Discussion
Limitations	19	Trustworthiness and limitations of findings	4.1, Limitations and Future Research Directions

**Other**			
Conflicts of interest	20	Potential sources of influence or perceived influence on study conduct and conclusions; how these were managed	Conflict of Interest
Funding	21	Sources of funding and other support; role of funders in data collection, interpretation, and reporting	Funding
